# Individual point mutations in two ERAD E2 ubiquitin-conjugating enzymes do not affect *Caenorhabditis elegans* spontaneous reversal frequency

**DOI:** 10.17912/micropub.biology.000328

**Published:** 2020-11-19

**Authors:** Mackenzi Oswald, Heino Hulsey-Vincent, Caroline (Lina) Dahlberg

**Affiliations:** 1 Department of Biology, Western Washington University, Bellingham, WA, 98225, USA

**Figure 1. Mutations in E2 ubiquitin-conjugating enzyme genes and glr-1 affect spontaneous reversal frequencies differently:**
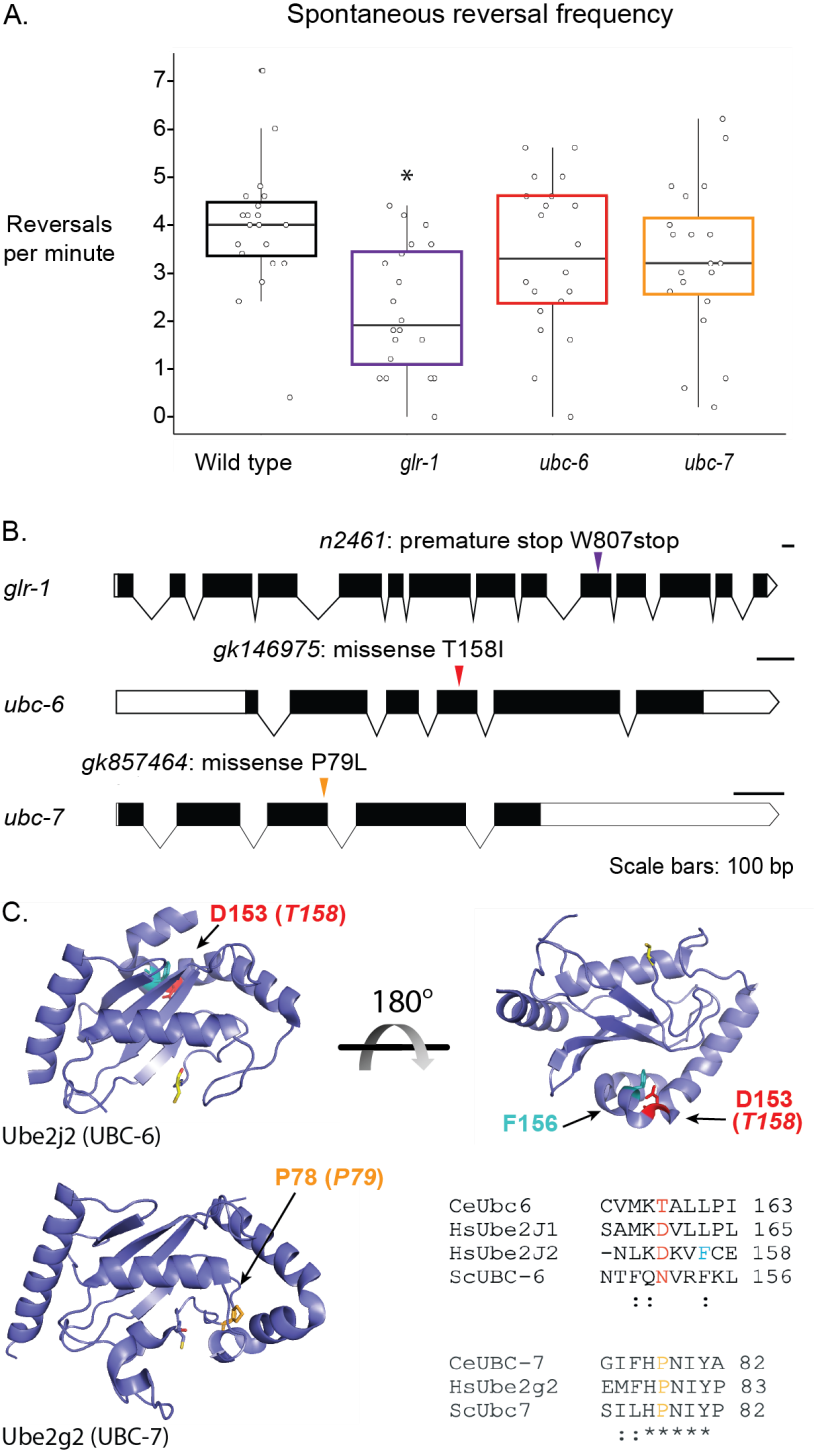
**A.** Spontaneous reversal frequency assays were performed using wild type (WT) *C. elegans*, alongside strains harboring mutations in *glr-1* or in genes that encode the E2 ubiquitin-conjugating enzymes, *ubc-6* and *ubc-7*. Box and whisker plots show the average reversals per minute, bounded by quartiles; the line in each box represents the median of the average reversals per minute for each genotype. N=20 individual animals for all genotypes; significance relative to WT was calculated using the Tukey-Kramer test following a one-way ANOVA (*, p=0.0027 for *glr-1*; non-significant: p=0.58 for *ubc-6*; p=0.48 for *ubc-7*). The data is normally distributed (Shapiro-Wilk test, p=0.86) and groups show equal variance (residuals vs. fitted plot). **B.** Schematics of E2 ubiquitin-conjugating enzyme genes and *glr-1* gene, including the locations of point mutations. Schematics were made using http://wormweb.org/exonintron. **C.** Crystal structures of the human E2 Ube2j2 (top, with rotated view to show the back side of the protein) and Ube2g2 (bottom, left). Models were made using Pymol. Residues that are mutated are shown in red (Ube2j2, D153; UBC-6, T158) and yellow (Ube2g2, P78, UBC-7, P79). Residue F156 of Ube2j2 is shown in cyan. Alignments of *C. elegans* UBC-6 and UBC-7 regions of homology, compared to putative orthologs in other eukaryotes. The highlighted residues correspond to those shown in the structures.

## Description

Maintaining proteostasis, or protein homeostasis, is an important cellular function because misfolded proteins can aggregate and contribute to neurodegenerative diseases. One way that cells preserve proteostasis is through the Endoplasmic Reticulum Associated Degradation pathway (ERAD). ERAD relies on interactions between E2 ubiquitin-conjugating enzymes and E3 ubiquitin ligases to ubiquitylate misfolded proteins in order to signal for their destruction via the proteasome (Vembar and Brodsky 2008). We investigated if missense mutations in genes that encode two E2 ubiquitin-conjugating enzymes in *C. elegans*, UBC-6 and UBC-7, affect spontaneous reversal frequency (Brockie *et al.* 2001; Jones *et al.* 2002; Stewart *et al.* 2016; Zheng *et al.* 1999).

UBC-6 and UBC-7 are conserved across Eukaryotes (Figure 1C). We obtained strains of animals from the Million Mutation Project with the missense mutations that confer the amino acid changes T158I in UBC-6 and P79L in UBC-7. The analogous residues in human Ube2j1 or Ube2j2 and Ube2g2 are Asp160 or 153 and Proline 78, respectively, which were mapped onto the available structures of the human enzymes (Figure 1C). In Ube2j2 (*C. elegans* UBC-6) Asp153 makes contact with Phe156, and mutating this residue decreases the efficiency of the enzyme (Tobi Ritterhoff, personal communication). Structural alignments suggest that Pro78 (mutated in UBC-7) is part of the well-conserved HPN (Histadine-Proline-Asparagine) motif, so mutation of the analogous residue was hypothesized to abrogate enzymatic activity (Cook and Shaw 2012; Wu *et al.* 2003).

Our results show that the E2 ubiquitin-conjugating enzyme mutations did not significantly affect *C. elegans* spontaneous reversal frequency (Figure 1A). *glr-1* animals were used as a control and reversed significantly less frequently than wild-type animals, as previously reported (Kowalski *et al.* 2011). Both *ubc-6* and *ubc-7* animals had less frequent spontaneous reversals than WT animals and more frequent spontaneous reversals than *glr-1* animals, but neither of these results were significant.

One clear reason for the insignificant change in reversal frequency in the *ubc-6* and *ubc-7* mutant animals could be that the missense mutations in these strains we observed do not affect gene function. It is also possible that UBC-6 and UBC-7 are able to compensate for each other in *C. elegans.* In *S. cerevisiae* Ubc6p and Ubc7p work cooperatively and sequentially to target substrates in coordination with the E3 ligases Doa10p (orthologous to *C. elegans* MARC-6), and Hrd1p (orthologous to *C. elegans* HRD-1 and paralogous to HRDL-1) (Sasagawa *et al.* 2007; Weber *et al.* 2016). It could be that this mechanism of tandem ubiquitylation is not conserved in *C. elegans* despite its conservation for human UBE2J2 (Weber *et al.* 2016). The human orthologs HRD1 and UBE2J1 (an additional ortholog of Ubc6p) also work together to target substrate proteins for degradation (Bays *et al.* 2001; Burr *et al.* 2011). Therefore it is possible that *C. elegans* E3s could collaborate with either UBC-6 or UBC-7 to target substrate proteins for degradation. In the future, we plan to address these possibilities using double *ubc-6*; *ubc-7* point mutants and CRISPR-Cas9-targeted deletion mutants for UBC-6 and UBC-7, in combination with E3 ligase mutants.

## Methods

*Reversal assay protocol*: Each *C. elegans* strain was grown on separate NGM agar plates seeded with OP50, at 21.4°C. While setting up for the reversal assays, multiple young adulthermaphroditic nematodes from each strain were picked onto separate seeded NGM agar plates and coded. This allows for blind analysis of the videotaped trials to occur when scoring the reversal assays, in order to reduce potential bias. During the assay, one animal at a time was picked using halocarbon oil (a non-food substance) from its coded seeded plate onto an unseeded NGM agar plate to induce food-seeking behavior. The animal was allowed to move around for two minutes before videotaping began. If the animal did not move away from its initial position after two minutes it was discarded and not counted in the data set. Each animal was recorded for five minutes and then discarded. At least one individual of each strain was observed during each experimental session in order to allow for any potential variations in temperature and humidity to be accounted for equally across all strains. The total N values listed represent measurements from multiple experimental sessions.

*Scoring reversals*: After finishing recording all reversal assay trials for an experiment, the coded trials were viewed and scored blindly by one researcher. Movement was counted as a reversal if the tip of the nose moved backwards for at least 1/6th of the animal’s body length. The position of the posterior pharyngeal bulb was used as a marker for that distance.

*Recording setup*: An Olympus SZ61 microscope attached to a TLB 4000 Series Substage Illuminator base was used to record the videos. The microscope was connected to The Imaging Source DFK 31AF03 color camera, which was connected to a computer running Windows OS. The software used to record the videos was Debut Professional by NCH Software.

*Genotyping*: The genotypes of all strains were confirmed after completing the trials. The genotypes of KP4, CLD41 and CLD43 were confirmed using PCR and DNA sequencing.

Additional point mutations from the MMP were considered for these experiments (http://genome.sfu.ca/gexplore/) but only one other available allele (*ubc-6* (gk549681)) was also predicted to have an effect on enzyme function (Tobi Ritterhoff, personal communication). Although that strain was viable, we were unable to backcross it to remove background mutations. Other existing deletion mutants remove sequences outside of the coding locus of each gene. We also note that following our experiments, CRISPR-Cas9-targeted deletion mutants became available for each gene, but these were not available for use at the time of this study.

## Reagents

**Table 1**. *C. elegans* strains used.

**Table:** 

Strain Name	Genotype	Description	Reference
N2		Wild-type	
KP4	*glr-1(n2461)* III	*glr-1* putativeknockout, nonsense point mutation	Hart AC *et al.* 1995
CLD41	*ubc-6(gk146975)* II*5	E2 ubiquitin- conjugating enzyme putative reduction-of-function, missense mutation, backcrossed 5 times	Thompson O *et al.* 2013
CLD43	*ubc-7(gk857464)* III*5	E2 ubiquitin-conjugating enzyme putative reduction-of-function, missense mutation, backcrossed 5 times	Thompson O *et al.* 2013
